# Allelic Variation on Murine Chromosome 11 Modifies Host Inflammatory Responses and Resistance to *Bacillus anthracis*

**DOI:** 10.1371/journal.ppat.1002469

**Published:** 2011-12-29

**Authors:** Jill K. Terra, Bryan France, Christopher K. Cote, Amy Jenkins, Joel A. Bozue, Susan L. Welkos, Ragini Bhargava, Chi-Lee Ho, Margarete Mehrabian, Calvin Pan, Aldons J. Lusis, Richard C. Davis, Steven M. LeVine, Kenneth A. Bradley

**Affiliations:** 1 Department of Microbiology, Immunology, and Molecular Genetics, University of California at Los Angeles, Los Angeles, California, United States of America; 2 Bacteriology Division, U.S. Army Medical Research Institute of Infectious Diseases (USAMRIID), Fort Detrick, Frederick, Maryland, United States of America; 3 Department of Human Genetics, University of California at Los Angeles, Los Angeles, California, United States of America; 4 Department of Medicine, University of California at Los Angeles, Los Angeles, California, United States of America; 5 Department of Molecular and Integrative Physiology, University of Kansas Medical Center, Kansas City, Kansas, United States of America; The University of Texas-Houston Medical School, United States of America

## Abstract

Anthrax is a potentially fatal disease resulting from infection with *Bacillus anthracis*. The outcome of infection is influenced by pathogen-encoded virulence factors such as lethal toxin (LT), as well as by genetic variation within the host. To identify host genes controlling susceptibility to anthrax, a library of congenic mice consisting of strains with homozygous chromosomal segments from the LT-responsive CAST/Ei strain introgressed on a LT-resistant C57BL/6 (B6) background was screened for response to LT. Three congenic strains containing CAST/Ei regions of chromosome 11 were identified that displayed a rapid inflammatory response to LT similar to, but more severe than that driven by a LT-responsive allele of the inflammasome constituent NRLP1B. Importantly, increased response to LT in congenic mice correlated with greater resistance to infection by the Sterne strain of *B. anthracis*. The genomic region controlling the inflammatory response to LT was mapped to 66.36–74.67 Mb on chromosome 11, a region that encodes the LT-responsive CAST/Ei allele of *Nlrp1b*. However, known downstream effects of NLRP1B activation, including macrophage pyroptosis, cytokine release, and leukocyte infiltration could not fully explain the response to LT or the resistance to *B. anthracis* Sterne in congenic mice. Further, the exacerbated response in congenic mice is inherited in a recessive manner while the *Nlrp1b-*mediated response to LT is dominant. Finally, congenic mice displayed increased responsiveness in a model of sepsis compared with B6 mice. In total, these data suggest that allelic variation of one or more chromosome 11 genes in addition to *Nlrp1b* controls the severity of host response to multiple inflammatory stimuli and contributes to resistance to *B. anthracis* Sterne. Expression quantitative trait locus analysis revealed 25 genes within this region as high priority candidates for contributing to the host response to LT.

## Author Summary

We show that genetic variation within an 8.3 Mb region on mouse chromosome 11 controls host response to anthrax lethal toxin (LT) and resistance to infection by the Sterne strain of *Bacillus anthracis*. Specifically, congenic C57BL/6 mice in which this region of chromosome 11 is derived from a genetically divergent CAST/Ei strain presented with a rapid and strong innate immune response to LT and displayed increased survival following infection with Sterne spores. CAST/Ei chromosome 11 encodes a dominant LT-responsive allele of *Nlrp1b* that may partially account for the severe response to LT. However, the strength of this response was attenuated in mice with only one copy of chromosome 11 derived from CAST/Ei indicating the existence of a recessive modifier of the inflammatory response to LT. In addition, congenic mice displayed a pronounced immune response using an experimental model of sepsis, indicating that one or more genes within the chromosome 11 region control host response to multiple inflammatory stimuli. Analyzing the influence of allelic variation on gene expression identified 25 genes as candidates for controlling these responses. In summary, we report a genetic model to study inflammatory responses beneficial to the host during anthrax.

## Introduction

Microbial pathogens have evolved various mechanisms to block host immune responses and thereby increase virulence. The MAP kinase (MAPK) signaling pathways have a central role in innate immune responses mounted by both plants and animals, and are common targets that are inactivated by a variety of bacterial toxins and effector molecules [1], [2]. *B. anthracis* produces anthrax lethal toxin (LT), a bipartite toxin that contributes to immunosuppression and pathology in the host [3]. The catalytic moiety of anthrax LT, lethal factor (LF), is a zinc-dependent metalloprotease that cleaves the N-termini of MAPK kinases (MKKs). By inactivating MKKs, LT blocks production of proinflammatory chemokines and cytokines such as TNF-α and inhibits survival signals activated via downstream MAPKs [4]–[11]. Thus, LT-mediated cleavage of MKKs leads to the silencing of a pro-inflammatory response, effectively repressing host immunity and favoring bacterial survival [9], [12], [13].

In response to such pathogenic mechanisms, eukaryotic hosts have evolved means to detect and counter pathogen encoded virulence factors that target intracellular signaling pathways. Specifically, nucleotide-binding domain leucine-rich repeat (NLR) proteins sense bacterial products or host cell-derived danger signals to initiate defense pathways. NLR-mediated responses can function locally through induction of cell death and/or distally through production and release of antimicrobial products and signaling molecules. Allelic variation at the NLR gene, *Nlrp1b,* in rodents is one mechanism that controls the host cellular response to LT and subsequent sensitivity to *B. anthracis* infection [14]–[16]. Specifically, LT-responsive alleles of *Nlrp1b* drive caspase-1 mediated proinflammatory cell death, termed pyroptosis, of macrophages and dendritic cells. Increased resistance to *B. anthracis* is correlated with LT activation of the NLRP1B inflammasome, resulting in IL-1β release and pyroptosis [15], [17].

Sensitivity of multiple animal species to anthrax varies inversely with sensitivity to injection of purified LT [18]. This inverse relationship holds true when comparing inbred strains of mice [19]. Therefore, genetic comparison of mouse strains is predicted to reveal mechanisms of the host response to *B. anthracis*. Indeed, differential sensitivity of mouse strains to *B. anthracis* infection is known to be influenced by allelic variations in at least two genes: *Nlrp1b* and *Hc* encoding complement C5 [15], [17], [19]–[23]. Allelic variation of these genes does not, however, fully account for differential sensitivity to infection or to intoxication by LT [17], [24]. Therefore, we hypothesized that additional genes may contribute to host susceptibility to anthrax. Due to the critical role of LT as a virulence determinant for *B. anthracis*, a genome-wide collection of congenic mouse strains was screened for altered responses to this toxin. Here we report the identification of a quantitative trait locus (QTL) on chromosome 11 that influences host response to multiple inflammatory stimuli including LT, resulting in increased resistance to *B. anthracis* Sterne infection.

## Results

### CAST/Ei Alleles on Chromosome 11 Are Associated with a Rapid and Severe Inflammatory Response to LT and Endotoxin

**Figure 1:**
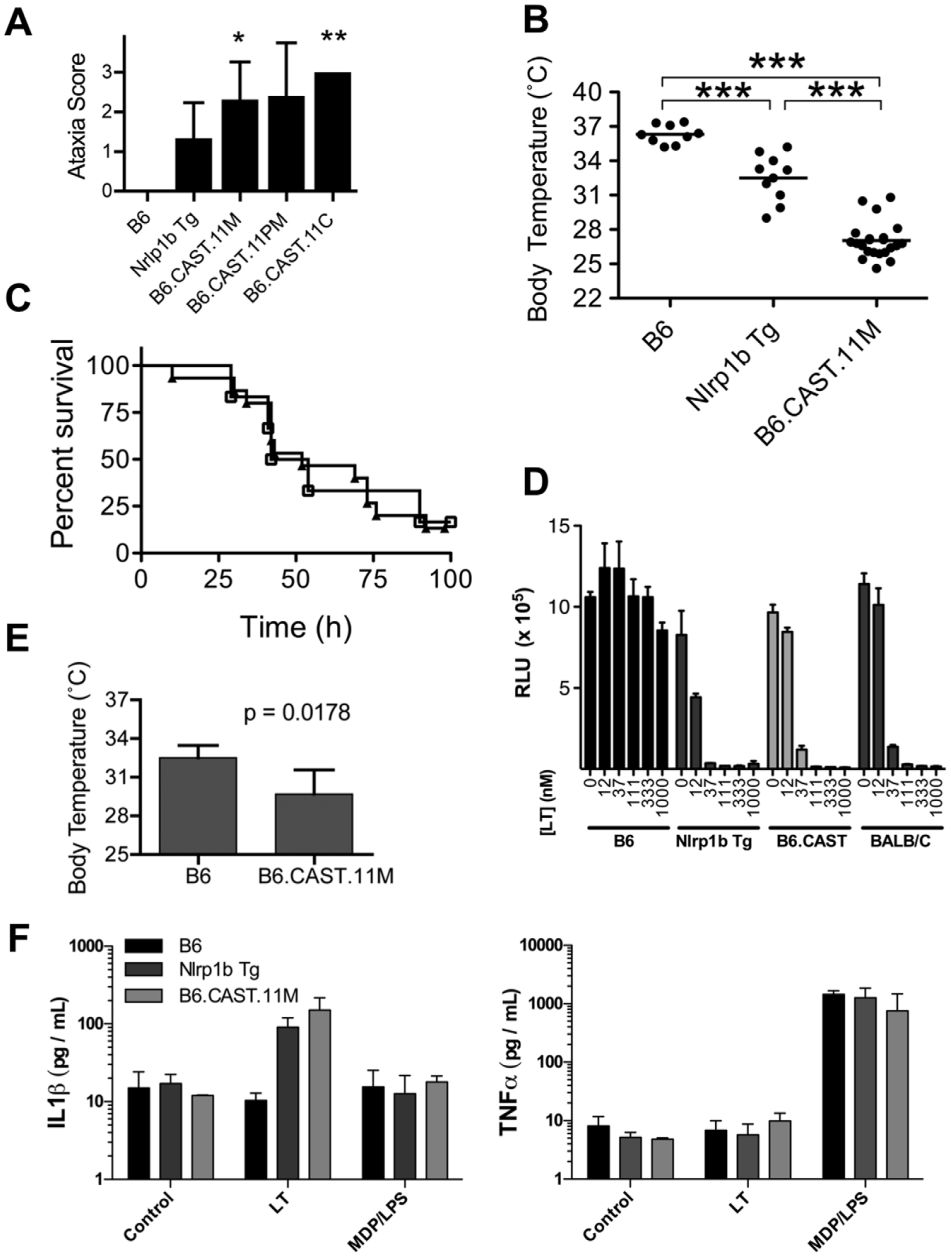
Identification of severe early response phenotype in B6.CAST.11 mice. B6.CAST.11M mice (n = 16), B6.CAST.11PM mice (n = 5), B6.CAST.11C mice (n = 5), mice expressing a LT-sensitive allele of Nlrp1b (B6*^Nrlp1b^* ^(129S1)^) (n = 15), or control B6 animals (n = 6) were injected i.p. with 5 µg PA+2.5 µg LF per g body weight and were scored for ataxia severity (**A**) and survival (**C**). **A,** Animals were scored on a 0–3 scale in which numbers represent the following walking ataxia scores: 0 =  no ataxia; 1 =  mild; 2 =  moderate; 3 =  severe, immobile, or moribund. See Materials and Methods for the definition of the ataxia scoring scale. The highest ataxia score during 7 h post injection is plotted +/− SD. *P* values calculated by Kruskal-Wallis nonparametric test with Dunn's post test. *p<0.05; **p<0.01 compared with B6. **B,** B6.CAST.11M (n = 22), B6*^Nrlp1b^* ^(129S1)^ (n = 10), or control B6 animals (n = 9) were challenged i.p. with 15 µg PA+7.5 µg LF per g body weight (equivalent in activity to dose used in **A**, **C**) and the lowest temperature observed during the first 5 h post toxin injection is plotted. ***p<0.001 calculated by 1-way ANOVA with Tukey post test. **C,** Animals were monitored for presentation of clinical signs associated with terminal LT-induced disease and euthanized upon reaching a moribund state. Data represent time to euthanasia following LT injection. Symbols represent: squares, B6 (n = 6); triangles, B6.CAST.11M (n = 16). **D,** BMDMs derived from the indicated mouse strains were seeded in 96-well plates and treated with increasing concentrations of PA and LF for 4 h. Viability was assessed using ATPlite 1-step reagent (Perkin Elmer). Average luminescence values of triplicate samples are graphed +/− SEM. **E,** Mice (n = 5 / group) were injected i.p. with 5 µg/g MDP 4 h prior to i.p. challenge with 1 µg/g LPS. Rectal temperature was then measured hourly, and the lowest body temp achieved by each animal within 4 h is graphed +/− SD. *P*-value was calculated from unpaired two-tail t-test. **F.** BMDMs derived from the indicated mouse strains were seeded in 96-well plates then challenged with 250 ng/mL LT for 3 h or 0.1 µg/mL MDP +0.1 ng/mL LPS for 8 h. Culture supernatants were then analyzed for IL-1β and TNFα as described in methods. Data represent average values +/− SD.

To identify chromosomal regions affecting response to LT, a library of congenic mice consisting of homozygous CAST/Ei segments on a B6 background was screened. The genome coverage of this library spans roughly 80% of the autosomal chromosomes and consists of approximately three strains per chromosome in which CAST/Ei segments are introgressed onto the C57BL/6J (B6) background in an overlapping manner [25]. Three strains, B6.CAST.11 medial (B6.CAST.11M), B6.CAST.11 proximal medial (B6.CAST.11PM), and B6.CAST.11 complete (B6.CAST.11C), all harboring CAST/Ei segments on chromosome 11, displayed a rapid and transitory response following LT injection similar to the early response phenotype (ERP) previously observed in LT-challenged B6*^Nlrp1b^* ^(129S1)^ transgenic mice [15]. The latter express a 129S1/SvImJ(129S1)-derived LT-responsive allele of *Nlrp1b* on an otherwise LT-resistant B6 background. Akin to the ERP of LT-injected B6*^Nlrp1b^* ^(129S1)^ transgenic mice, chromosome 11 B6.CAST mice presented with ataxia (**Figure 1A** **)**, hypothermia (**Figure 1B**) and one or more of the following: bloat, dilated vessels on pinnae, loose/watery feces, labored abdominal breathing (not shown). This response developed as early as 30 min post LT injection, and all animals presented by 4 h (not shown). Importantly, the ERP displayed by chromosome 11 B6.CAST mice was significantly more pronounced compared to that in B6*^Nlrp1b^* ^(129S1)^ animals as evidenced by a more severe ataxia score (**Figure 1A**) as well as a more severe hypothermic state (**Figure 1B**). Other congenic strains and B6 mice did not display clinical signs associated with the ERP (data not shown). Upon careful observation, CAST/Ei and BALB/c strains displayed a very mild, inconsistent version of these early signs (data not shown), indicating that the mixture of CAST/Ei alleles of chromosome 11 genes with B6 alleles accounting for the rest of the genome likely resulted in increased expressivity of a toxin-responsive phenotype present in LT-responsive strains. The response to LT was independent of route of toxin administration and was present when mice were administered toxin intravenously (i.v.) (not shown). Interestingly, i.v. LT challenge produced an accelerated presentation of the ERP that was observed as quickly as 18 min post challenge (not shown). Following the ERP, chromosome 11 B6.CAST mice typically recovered to normal behavior within 4–25 h post LT injection and subsequently relapsed into a second round of clinical signs, eventually succumbing to moribund state and/or death within the same timeframe as parental B6 mice (**Figure 1C**, and not shown). Endotoxin contamination of protective antigen (PA), the host cell-binding moiety of LT, or LF was not responsible for the ERP or ultimate lethality, as no response was detected following injection of a 2X dose of individual toxin components (data not shown).

Anthrax LT induces a rapid pyroptotic cell death in macrophages derived from mice with LT-responsive alleles of *Nlrp1b.* Five murine alleles of *Nlrp1b* have been described [14]. Allele 2, encoded by B6 mice, and alleles 3 and 4 do not respond to LT, while allele 1, encoded by 129S1 and Balb/C mice, and allele 5, encoded by CAST/Ei mice, are LT-responsive. Responsiveness to LT is fully dominant and macrophages from heterozygous mice with one LT-responsive and one LT-resistant allele display sensitivity to LT indistinguishable from macrophages encoding two LT-responsive alleles ([26] and data not shown). The kinetics of ERP in mice is consistent with timing of macrophage and DC pyroptosis treated with LT *ex vivo*. Therefore, we sought to determine whether allelic variation of *Nlrp1b* influenced sensitivity of macrophages to LT. Bone marrow derived macrophages (BMDMs) from B6*^Nlrp1b^* ^(129S1)^ and B6.CAST.11M mice, as well as those from BALB/c mice encoding an LT-sensitive allele of *Nlrp1b* (allele 1) displayed similar sensitivity to LT *ex vivo* (**Figure 1D**). Further, BMDMs from B6*^Nlrp1b^* ^(129S1)^ and B6.CAST.11M mice displayed similar IL-1β responses to LT (**Figure 1F**). Together, these data demonstrate that the more severe ataxia and hypothermia observed in B6.CAST.11M mice did not result from alterations in LT-induced macrophage pyroptosis.

We next tested whether B6.CAST.11M mice have an altered response to additional inflammatory stimuli. Preliminary studies indicated that B6.CAST.11M mice, but not B6 controls, display ataxia and clinical signs associated with inflammation following challenge with recombinant IL-1β (not shown). To further text LT-independent inflammatory responses, an established model of sepsis was employed whereby mice were injected i.p. with muramyl dipeptide (MDP) and lipopolysaccharide (LPS), resulting in a rapid TNFα-dependent hypothermia [27]. Using this model, B6.CAST.11M mice displayed exacerbated ataxia (not shown) and hypothermia (**Figure 1E**) compared to B6 control animals. This response was not due to alterations in macrophage responsiveness to MDP/LPS as determined by TNFα and IL-1β release (**Figure 1F**). These results are consistent with a heightened responsiveness of CAST/Ei chromosome 11 alleles to multiple inflammatory stimuli.

**Figure 2:**
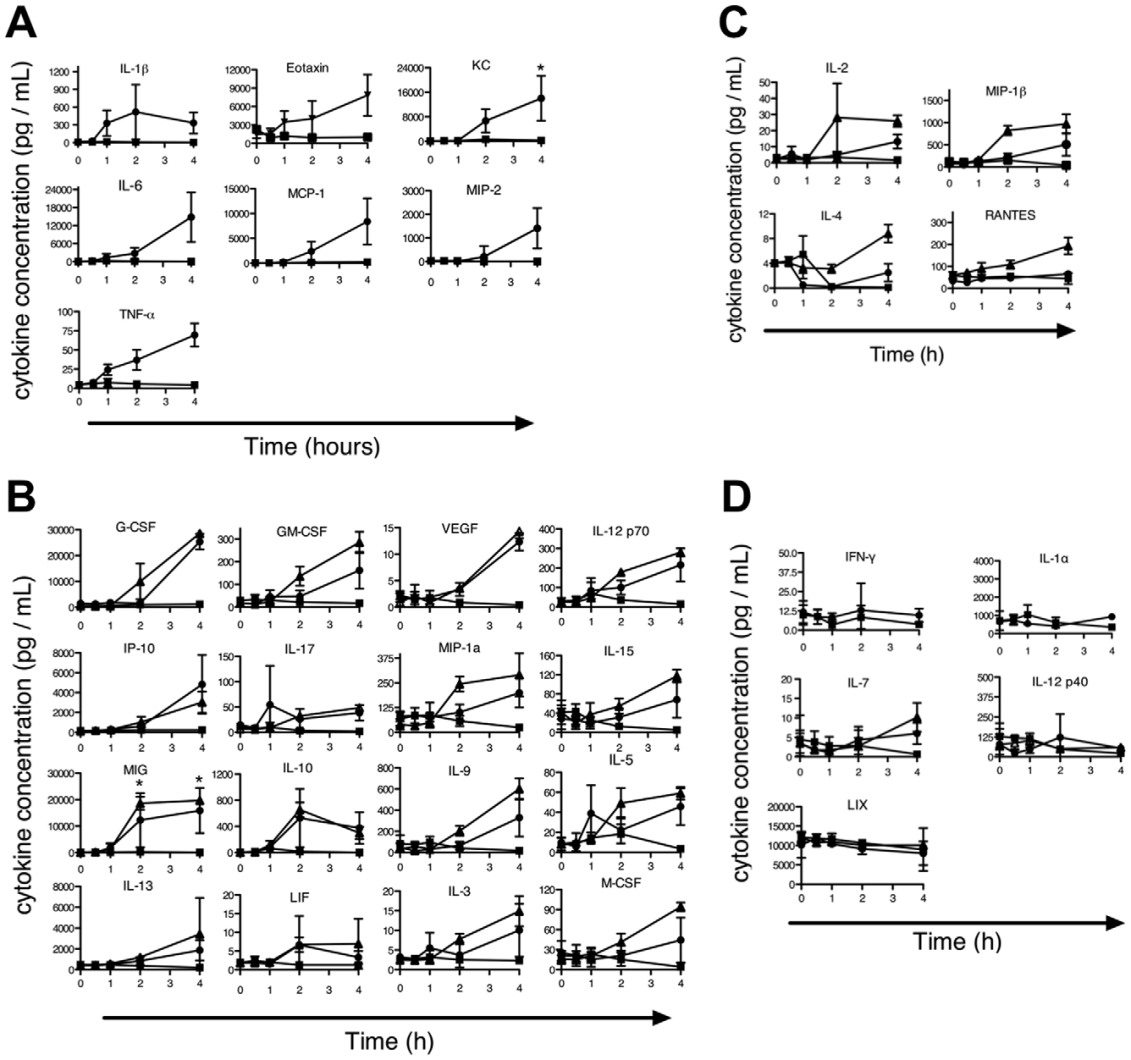
Evaluation of cytokine profile during the LT-induced Early Response Phenotype. B6.CAST.11M (circles), B6*^Nrlp1b^* ^(129S1)^ (triangles), or nontransgenic littermate control mice (squares) were challenged with LT as in Figure 1B. Uninjected animals served as t = 0 controls. Animals were sacrificed at 0.5, 1, 2, and 4 h post toxin injection, and serum cytokines levels were measured. Data represent the average values of five animals per group +/− SD. **A**–**B,** Several proinflammatory cytokines were induced in B6.CAST.11M and B6*^Nrlp1b^* ^(129S1)^ mice that were not induced in nontransgenic B6 control animals. See [15] for B6*^Nrlp1b^* ^(129S1)^ responses for cytokines in (A). **C**, Cytokines differentially induced in B6*^Nrlp1b^* ^(129S1)^ mice compared to B6.CAST.11M mice. **D,** Cytokines not induced in either B6.CAST.11M or B6*^Nrlp1b^* ^(129S1)^ mice.

Previously, we reported that the LT-induced ERP in B6*^Nlrp1b^* ^(129S1)^ mice was associated with release of proinflammatory cytokines [15]. To determine whether a proinflammatory cytokine response also accompanies the LT-induced ERP phenotype in chromosome 11 B6.CAST mice, sera from LT-challenged animals were analyzed. As predicted, proinflammatory cytokines previously identified as induced in B6*^Nlrp1b^* ^(129S1)^ mice were also induced in B6.CAST.11M mice following LT challenge (**Figure 2A**). Interestingly, these cytokines were induced to a similar level and with similar kinetics in both strains despite the exacerbated ERP displayed by B6.CAST.11M mice compared to B6*^Nlrp1b^* ^(129S1)^ [15]. This finding is consistent with the observation that BMDMs derived from these strains displayed similar pyroptotic responses to LT (**Figure 1D, F**). Endotoxin contamination of PA or LF was not responsible for the cytokine induction observed, as no response was detected following injection of a 2X dose of individual toxin components (not shown). To further test whether alterations in cytokine responses could explain the altered phenotype severity, a panel of additional cytokines and chemokines were assayed following LT challenge (**Figure 2B**–**D**). A total of 27 cytokines were induced in B6.CAST.11M and/or B6*^Nlrp1b(129S1)^* but not B6 mice (**Figure 2A** –**C**), while five cytokines showed no response in any strain (**Figure 2D**). Only four out of 27 cytokines that responded to LT were differentially induced in the sera from B6*^Nlrp1b^* ^(129S1)^ mice compared to sera from B6.CAST.11M mice (**Figure 2C**). Interestingly, all four of these cytokines were preferentially induced in B6*^Nlrp1b^* ^(129S1)^ mice compared to B6.CAST.11M mice. Of the cytokines differentially induced, three function as pro-inflammatory mediators while one cytokine, IL-4, exhibits both pro- and anti-inflammatory properties [28].

### Role of IL-4 and IL-1 in ERP

**Figure 3:**
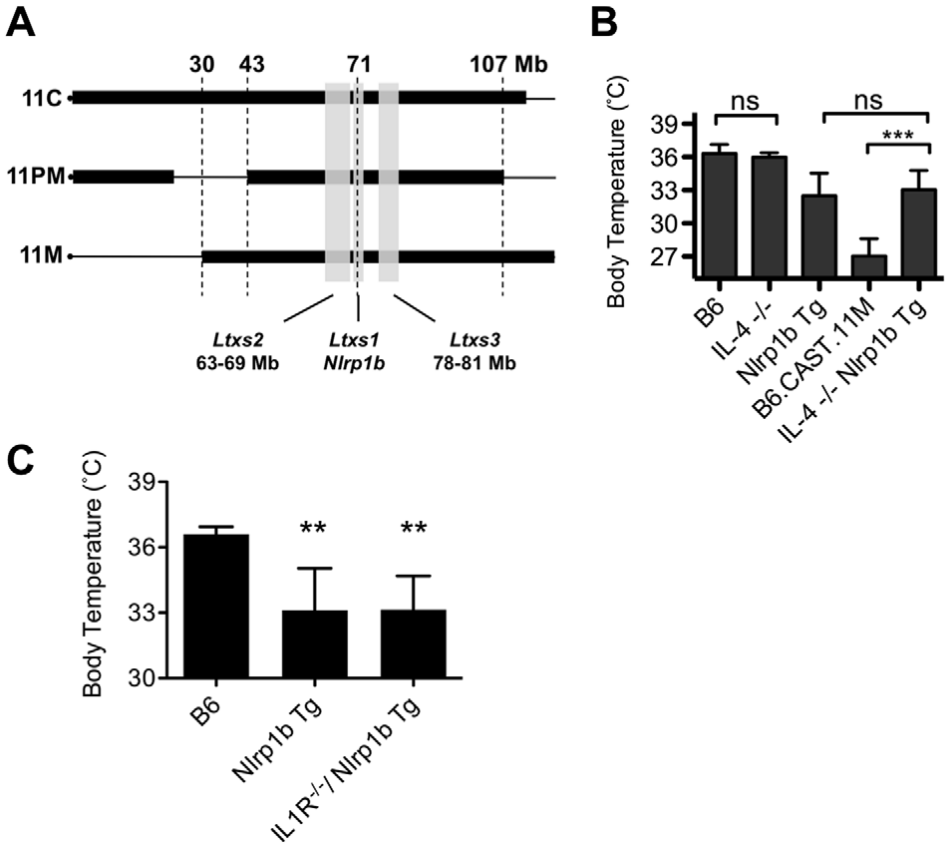
The role of IL-4 in the Nlrp1b-mediated response to LT. **A,** Comparative maps of mouse chromosome 11 B6.CAST strains. Thin horizontal lines indicate homozygous B6 sequence, while thick boxes indicate homozygous CAST/Ei sequence. Physical locations are shown on top. Vertical gray boxes denote QTL *Ltxs1-3*. Locations are as follows (physical / recombination boundaries): *Ltxs2* = 63–69 Mb / 34.5 - 37 cM; *Ltxs1* = 69.5–71 Mb / 40–43 cM; *Ltxs3* = 78–81 Mb / 44–46 cM. **B**, Mice deficient for the *IL4* gene and expressing a LT-sensitive allele of *Nlrp1b* (B6*^Nlrp1b^* ^(129S1)^/*IL4* ^−/−^) (n = 7), mice deficient for the *IL4* gene and expressing a LT-resistant allele of Nlrp1b (*IL4* ^−/−^) (n = 7) were injected i.p. with 15 µg PA+7.5 µg LF per g body weight, and body temperatures were monitored hourly. The lowest temperature observed during the first 6 h post toxin injection is plotted as mean values +/− SD. Data for controls (B6, B6*^Nrlp1b^* ^(129S1)^ and B6.CAST.11M) are re-graphed from Figure 1B for comparison to IL-4^−/−^. Lack of *IL4* did not significantly alter response to LT compared to matched control groups. **C,** B6 (n = 5), B6*^Nlrp1b^* ^(129S1)^ (n = 5), and B6*^Nlrp1b^* ^(129S1)^ *IL-R* ^−/−^ (n = 10) mice were challenged i.p. with 15 µg PA+7.5 µg LF per g body weight and the lowest temperature observed during the first 6 h post toxin injection is plotted +/− SD. Lack of *IL1R* did not significantly alter response to LT in mice transgenic for *Nlrp1b.* **p<0.01 compared to B6 control.

B6.CAST congenic strains displaying the LT-mediated ERP share a CAST/Ei derived critical region between 43–107 Mb on chromosome 11 (**Figure 3A**). A single cytokine, IL-4, preferentially induced in B6*^Nlrp1b^* ^(129S1)^ mice during the LT-induced ERP relative to B6.CAST.11M mice (**Figure 2C**) maps to this critical region and is encoded at 53.4 Mb on chromosome 11. Interestingly, IL-4 functions as a T_h_2 cytokine, and elevated expression of IL-4 has been linked to reduced inflammation during sepsis in humans [29]. To determine the role of IL-4 in the inflammatory response to LT, mice deficient in IL-4 but expressing a LT-responsive allele of *Nlrp1b* were generated and tested for their response to LT. If reduced IL-4 levels in B6.CAST.11M mice are responsible for the exacerbated LT-induced ERP, then B6*^Nlrp1b^* ^(129S1);*IL4*−/−^ mice would be predicted to display a strong ERP akin to that in B6.CAST.11M mice. However, B6*^Nlrp1b^* ^(129S1);*IL4*−/−^ mice displayed an ERP equal in strength to B6*^Nlrp1b^* ^(129S1)^ mice following LT injection (**Figure 3B**), indicating that the absence of IL-4 does not affect ERP severity following LT challenge.

Nlrp1b inflammasome activation results in ASC-dependent maturation of cytokines including IL-1β, as well as ASC-independent cell lysis [30]. The ERP is associated with high serum concentrations of multiple cytokines, of which IL-1β is one of the earliest detectable (**Figure 2A**). To determine whether IL-1β is required for the ERP, B6.CAST.11M, and B6*^Nlrp1b^* ^(129S1)^ mice were pretreated with a blocking antibody to IL-1β, then challenged with LT. Notably, this treatment had no significant effect on ERP as determined by ataxia scoring (not shown). However, it remained possible that the anti IL-1β antibody treatment was not sufficient to block the LT-induced release of this cytokine. Therefore, mice deficient in the type 1 IL-1 receptor (IL-1R) and expressing the LT-responsive 129S1 allele of *Nlrp1b* were tested for response to LT. Consistent with the antibody studies, IL-1R deficient B6*^Nlrp1b^* ^(129S1)^ animals displayed ataxia (not shown) and hypothermic responses indistinguishable from B6*^Nlrp1b^* ^(129S1)^ mice (**Figure 3C**).

### B6.CAST. 11M Mice Display Resistance to Infection by *Bacillus anthracis* Sterne Strain

**Figure 4:**
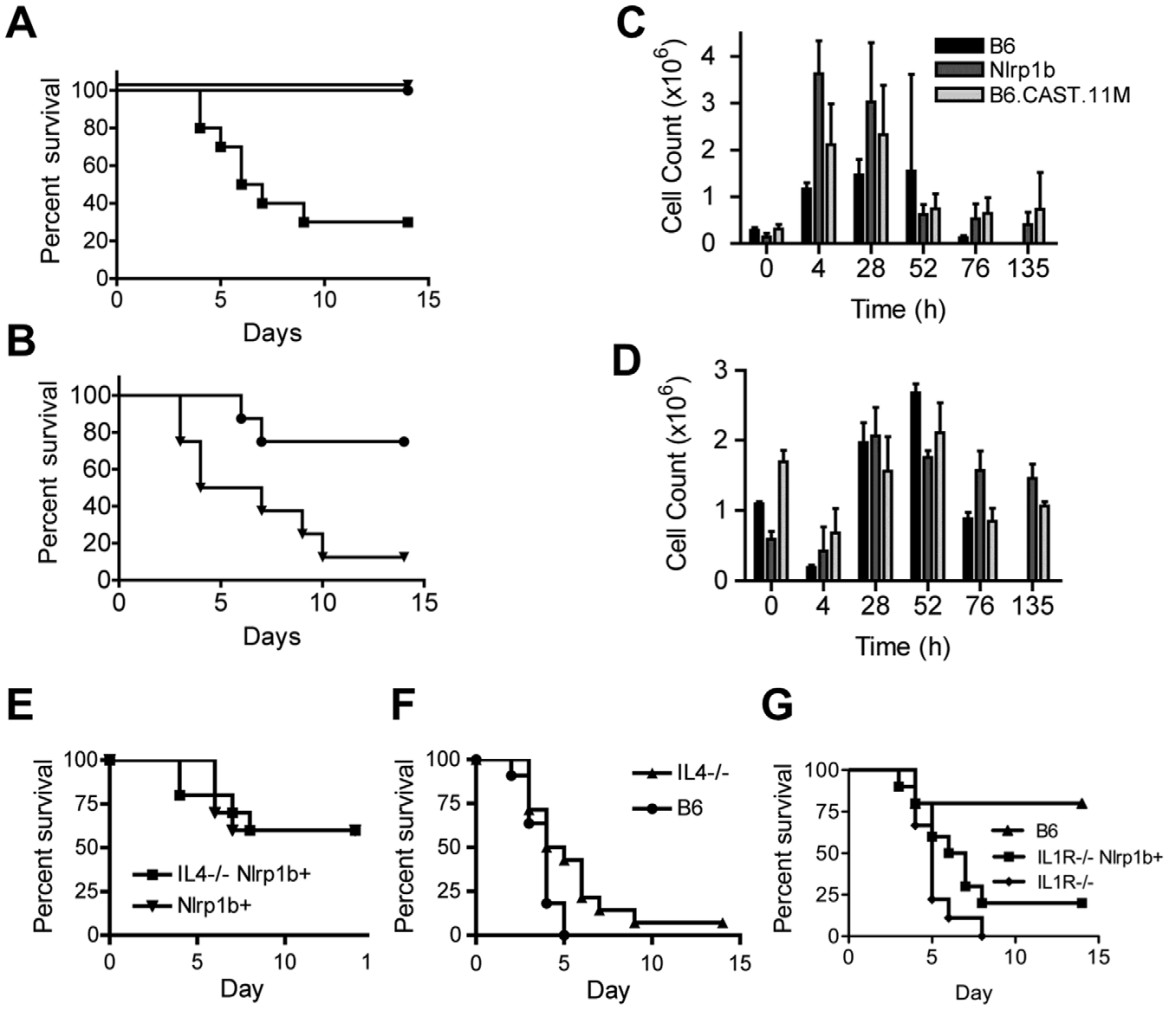
B6.CAST.11M mice display increased resistance to infection by *B. anthracis* Sterne. **A**, Kaplan-Meier analysis of B6.CAST.11M (n = 8)(circles), B6*^Nrlp1b^* ^(129S1)^ (n = 8)(triangles), or nontransgenic littermate control mice (n = 8)(squares) challenged i.p. with 3×10^7^ spores of *B. anthracis* Sterne strain 7702. Survival curve p<0.0001 for both experimental groups compared to B6 control. **B**, Kaplan-Meier analysis of B6.CAST.11M (n = 8)(circles) or B6*^Nrlp1b^* ^(129S1)^ (n = 8)(triangles) challenged i.p. with 4×10^8^ spores of *B. anthracis* Sterne strain 7702. Survival curve p = 0.0126. **C & D,** For cellular analyses at infection site following spore challenge, animals were euthanized at the indicated time points and the number of PMNs (**C**) and monocytes (**D**) in the peritoneal cavity were determined as described in the Materials and Methods section. Data represent mean values (n = 3 except, n = 2 for B6.CAST.11M at 72 h and B6*^Nrlp1b^* ^(129S1)^ at 135 h, and n = 0 for B6 at 135 h) ± SD. Differences in PMN counts were significant as indicated: [t = 4 h, p = 0.025 B6.CAST.11M vs B6; p = 0.003 B6*^Nrlp1b^* ^(129S1)^ vs B6; p = 0.04 B6.CAST.11M vs B6*^Nrlp1b^* ^(129S1)^] [t = 28, p = 0.016 B6*^Nrlp1b^* ^(129S1)^ vs B6; p = 0.038 B6.CAST.11M vs B6]. Data for B6 animals at 135 h is absent as no B6 mice survived until this time point. **E,** Kaplan-Meier analysis of B6*^Nlrp1b^* ^(129S1)^/*IL4* ^−/−^ (n = 10) and B6*^Nlrp1b^* ^(129S1)^ (n = 10) mice challenged i.p. with 1.14×10^8^ spores of *B. anthracis* Sterne strain 7702. **F,** Kaplan-Meier analysis of *IL4* ^−/−^ (n = 14) and B6 animals (n = 11) challenged i.p. with 2.42×10^7^ spores of *B. anthracis* Sterne strain 7702 (right panel). Survival curve, p = 0.033 **G,** Kaplan-Meier analysis of *IL1R* ^−/−^ mice with (n = 10) and without (n = 9) LT-sensitive 129S1 allele *Nlrp1b* challenged with 1.8×10^6^ spores of *B. anthracis* Sterne strain 7702. Animals were monitored for 14 days for survival.

Presentation of the ERP in response to LT correlates with an increased resistance to *B. anthracis* Sterne infection [15]. To determine whether the more severe ERP displayed by chromosome 11 B6.CAST mice correlates with an increased resistance to *B. anthracis* spore challenge, B6.CAST.11M, B6*^Nlrp1b^* ^(129S1)^, and B6 mice were challenged i.p. with 3×10^7^ Sterne strain spores. At this dose, all B6.CAST.11M and B6*^Nlrp1b^* ^(129S1)^ mice survived to the experimental endpoint whereas the majority of B6 animals succumbed to the infection (**Figure 4A**). Using a ∼13 fold higher dose of 4×10^8^ Sterne spores revealed that B6.CAST.11M mice were significantly more resistant to infection compared to B6*^Nlrp1b^* ^(129S1)^ mice (**Figure 4B**). Therefore, a more robust ERP correlates with increased protection from *B. anthracis* Sterne infection. Further, allelic variation of a chromosome 11– encoded gene(s) contributes to the increased ability of B6.CAST.11M congenic mice to limit *B. anthracis* Stern infection.

To determine the cellular mediators providing infection resistance, mice were challenged with Sterne spores i.p. and peritoneal exudates were collected and analyzed at various time points following challenge. A significant increase in the number of PMNs was observed in both B6*^Nlrp1b^* ^(129S1)^ and B6.CAST.11M mice at earlier time points following spore challenge compared to nontransgenic (i.e. B6) control mice (**Figure 4C**). These results correspond to previously reported data indicating a role for PMNs in bacterial clearance following *B. anthracis* spore challenge [12], [15], [21]. However, no significant differences were observed in the PMN response between B6*^Nlrp1b^* ^(129S1)^ and B6.CAST.11M mice that could explain their differential resistance to *B. anthracis*. Indeed, at 4 h, neutrophil influx in B6*^Nlrp1b^* ^(129S1)^ animals was significantly greater than that in B6.CAST.11M mice (p = 0.04). Similarly, monocytic infiltration could not explain the increased resistance seen in B6.CAST.11M mice (**Figure 4D**).

Given that leukocyte infiltration did not account for the difference in susceptibility to *B. anthracis*, we next considered whether the differential cytokine response could explain the increased resistance in B6.CAST.11M animals. Although IL-4 did not contribute to hypothermia (**Figure 3B**) or ataxia (not shown) following LT challenge, it remained possible that this cytokine still affected resistance to spore challenge. Indeed, as a T_h_2 cytokine, IL-4 can function by altering PMN and macrophage activity [31], [32]. Alteration of phagocyte function may contribute to differential responses in long-term studies such as resistance to spore challenge, but not contribute to immediate phenotypes such as ataxia and hypothermia following LT challenge (**Figure 3B**). To test the role of IL-4 in resistance to *B. anthracis*, B6*^Nlrp1b^* ^(129S1);*IL4*−/−^ mice were challenged i.p. with Sterne strain spores (**Figure 4E**). IL-4 deficiency did not affect host susceptibility to spore challenge in the presence of the LT-responsive allele of *Nlrp1b*, excluding this gene as a candidate. Interestingly, in the absence of an LT-responsive allele of *Nlrp1b*, the loss of IL-4 resulted in a slightly higher resistance to anthrax (**Figure 4F**).

Next, the role of IL-1β in *Nlrp1b*-mediated resistance to *B. anthracis* Sterne was tested. LT dampens the host cytokine response in the absence of LT-responsive *Nlrp1b* [9], [10], [33], [34]. However, this immunosuppression is not absolute and IL-1β contributes to resistance to *B. anthracis* even in the absence of LT-responsive *Nlrp1b*; animals expressing LT-resistant alleles of *Nlrp1b* and lacking IL-1R or MyD88 (required for TLR and IL-1R signaling) show increased sensitivity to infection by *B. anthracis* [35]–[38]. A critical role for IL-1R was further validated in mice expressing LT-responsive alleles of *Nlrp1b* [17]. However, it is still unknown whether increased resistance to *B. anthracis* mediated by LT-responsive alleles of *Nlrp1b* requires IL-1β. Of note, inflammasome-mediated resistance to *Francisella tularensis* is mediated by both IL-1β and IL-18, and mice deficient in either cytokine are resistant to tularemia, while those deficient in both IL-1β and IL-18 are sensitive [39]. To address the mechanism by which *Nlrp1b* mediates resistance to *B. anthracis*, B6*^Nlrp1b^* ^(129S1);IL-1R−/−^ mice were challenged with Sterne strain spores and viability was compared to that of spore-challenged B6^IL-1R−/−^ mice. Both strains showed similar susceptibility to *B. anthracis* (**Figure 4G**), indicating that *Nlrp1b-*mediated protection from anthrax requires IL-1β signaling.

### Severe LT-Induced ERP in B6.CAST.11 Mice Is Inherited in a Recessive Manner

**Figure 5:**
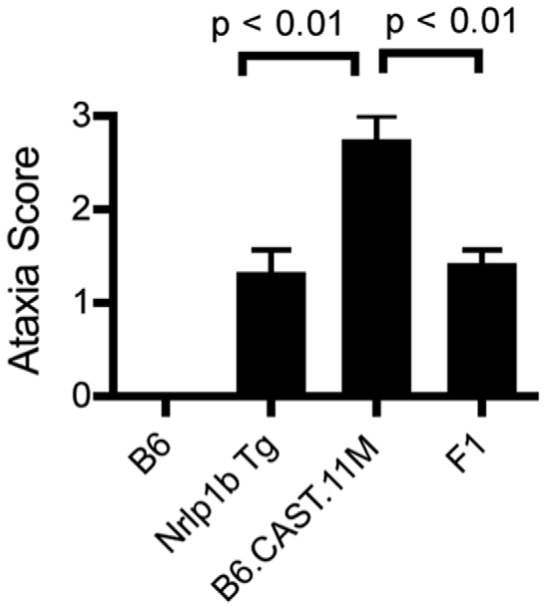
Severity of LT-induced ERP is controlled by a gene(s) on chromosome 11. **A,** B6.CAST.11M (n = 4), B6 (n = 4), B6*^Nrlp1b^* ^(129S1)^ (n = 14) and [B6.CAST.11M x B6] F_1_ (n = 14) animals were challenged i.p. with LT and responses scored as described above. Nonparametric analysis performed using Kruskal-Wallis nonparametric test with Dunn's post test.

We previously demonstrated that a LT-responsive 129S1 allele of *Nlrp1b* is sufficient to drive the ERP in B6*^Nlrp1b^* ^(129S1)^ mice [15]. The CAST/Ei allele of *Nlrp1b* is also LT-responsive, though distinct from the 129S1 allele [14]. To determine the contribution of the CAST/Ei allele of *Nlrp1b* in the B6.CAST.11 response to LT, B6.CAST.11M mice were crossed to B6 animals, and the resulting [B6.CAST.11M x B6] F_1_ mice were subsequently challenged with LT. LT-responsive alleles of *Nlrp1b* behave in a fully dominant and penetrant manner in controlling activation of caspase-1 and resulting macrophage pyroptosis [14], [26]. We therefore predicted that if allelic variation of *Nlrp1b* was responsible for the exacerbated ERP displayed by B6.CAST.11 mice, [B6.CAST.11M x B6] F_1_ offspring expressing one CAST/Ei allele of *Nlrp1b* would display an ERP equal in strength to the ERP presented by B6.CAST.11M mice. Strikingly, F_1_ animals displayed the ERP at a much weaker strength (**Figure 5**). Thus, the ERP observed in B6.CAST.11 mice is controlled by a gene or genes that behave in a recessive fashion.

### Critical Region Encoding Modifiers of the LT-Induced ERP Isolated to 66.36–74.67 Mb Region on Chromosome 11

**Figure 6:**
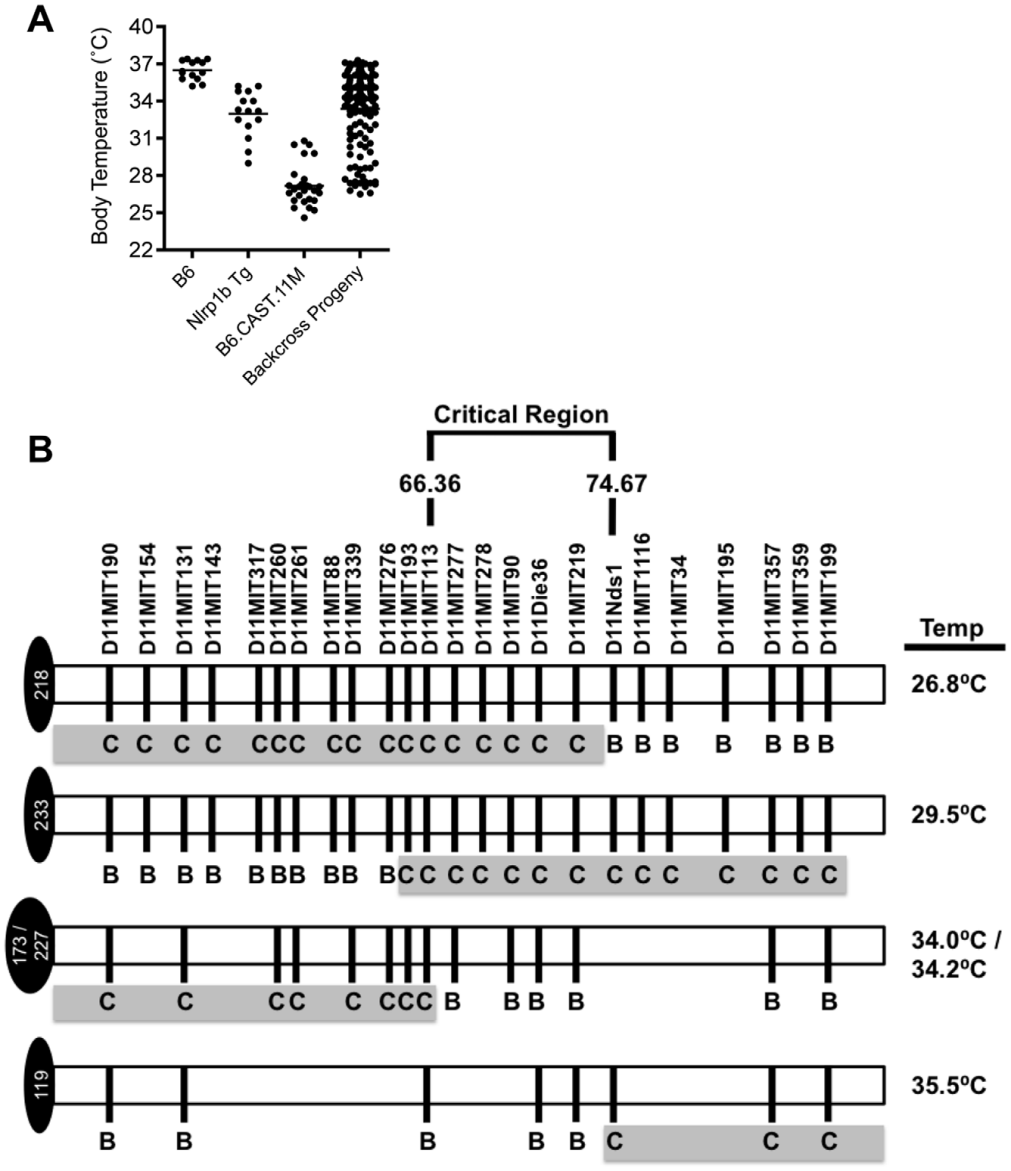
Refining the chromosome 11 critical region bestowing the LT-Induced ERP. **A**, Body temperature recordings of backcross progeny (n = 139) as well as B6.CAST.11M (n = 21), B6*^Nrlp1b^* ^(129S1)^ (n = 5) and B6 control mice (n = 5) following i.p. injection of 15 µg PA+7.5 µg LF. Mice were monitored as previously described. **B**, Backcross progeny genotypes at microsatellite markers on chromosome 11 were determined by PCR as described in Methods. Lowest body temperature for each animal following LT challenge was determined as described above.

To refine the critical region controlling the increased inflammatory response to LT, we undertook a positional cloning strategy. B6.CAST.11M mice were crossed to B6, and the resulting [B6.CAST.11M x B6] F_1_ mice were backcrossed to B6.CAST.11M animals. Backcross progeny (n = 139) were intoxicated and scored for ERP presentation based on ataxia and body temperature (**Figure 6A**). To correlate phenotype with genotype within the chromosome 11 critical region, backcross progeny were genotyped at multiple positions along the critical region using chromosome 11 microsatellite markers (**Figure 6B**). A subset of mice displaying a robust ERP following LT challenge, as evidenced by a body temperature less than 30°C and severe walking ataxia score, was heterozygous (i.e. CAST/Ei and B6) at multiple microsatellite markers localized within the original critical region on Chromosome 11 (**Figure 6B**). Conversely, several mice displayed a weak ERP, as evidenced by a body temperature greater than 34°C and mild walking ataxia score, despite retaining homozygous CAST/Ei alleles over a significant fraction of the critical region (**Figure 6B**). Comparison of the genotypes of these groups of mice revealed that a gene(s) that modifies host response to LT lies within the 66.36–74.67 Mb region on chromosome 11.

To identify candidate genes that contribute to host response to LT from the 273 genes in the current critical region, we employed expression quantitative trait locus (eQTL) analysis [40]–[42]. This approach relies on the hypothesis that differential gene expression is linked to phenotypic changes [40]–[43]. Transcript levels can be analyzed as genetic traits in the same way that phenotypes such as LT sensitivity can be analyzed [44], [45]. Using a single, large cross between two strains of mice, genetic loci, i.e. eQTL, that control differential transcript levels can be mapped. Importantly, data from a single experiment can be analyzed iteratively to identify a unique eQTL associated with every gene with an altered transcript level. If the transcript levels are controlled by structural variation of a gene that influences its rate of transcription or the maturation or stability of the transcript, the eQTL would be expected to map directly over the gene in question. Such an eQTL is termed a *cis*-acting eQTL. The approach of examining the segregation of transcript levels in a genetic cross should be distinguished from a study in which transcript levels are simply compared between two different strains. In the latter study, differences in transcript levels can be identified, but it is not possible to determine from such data whether any differences observed are the result of *cis*-acting genetic differences or *trans*-acting differences. In contrast, the combined genetic/gene expression approach makes it possible to identify genes and pathways that are perturbed by the *cis*- acting genetic variations.

Analysis of a dataset consisting of over 1,600 microarray experiments performed on 442F_2_ (B6 x CAST/EiJ) mice identified 81 *cis*-acting eQTL with a LOD score >4.3 within the refined critical region (**Table S1**). Comparison of genomic sequences of CAST/EiJ and B6 revealed multiple single nucleotide polymorphisms (SNPs) within the predicted mature mRNA sequences of 74 of the 81 eQTL, including non-synonymous changes in the coding regions (not shown). Further refinement based on reported gene function and expression pattern resulted in a list of 25 high-priority candidate genes (i.e., genes known to participate in immune or inflammatory processes) (**Table S1**).

## Discussion

We previously reported a role for a LT-sensitive *Nlrp1b* allele in controlling an inflammatory response to LT that we termed the ERP [15]. This response was characterized by the release of proinflammatory cytokines, noticeable ataxia and a reduction in body temperature that were not present in B6 animals encoding only LT-resistant alleles of *Nlrp1b* [15]. The ERP reported here in multiple B6.CAST.11 strains was more severe compared to the response exhibited by B6*^Nlrp1b^* ^(129S1)^ animals (**Figure** **1A, B**), and this correlated with increased resistance to *B. anthracis* (**Figure 4B**). Two models exist that may explain the altered response to LT between B6.CAST.11 and B6*^Nlrp1b^* ^(129S1)^ animals. First, variation between CAST/Ei and 129S1 alleles of *Nlrp1b* may directly contribute to ERP severity. Indeed, the current critical region contains *Nlrp1b*, and CAST/Ei alleles of this gene are LT-responsive yet genetically distinct from other LT-responsive alleles [14]. However, this explanation would require that CAST/Ei alleles of *Nlrp1b* contribute to phenotypes, such as response to MDP + LPS (**Figure 1E, F**), not currently associated with the Nlrp1b inflammasome. Further, two established mechanisms by which inflammasomes drive innate immune responses, macrophage pyroptosis (**Figure 1D** **)** and cytokine responses (**Figs. 1F** **,** **2**), cannot account for altered severity of host response to LT in B6.CAST.11 mice. Finally, [B6.CAST.11M x C57BL/6] F_1_ mice showed loss of the severe LT-induced ERP (**Figure 5**), indicating a recessive mode of inheritance that is inconsistent with the well-established dominance of LT-sensitive alleles of *Nlrp1b* [14], [26]. Interestingly, the ataxia scores in F_1_ mice match those seen in C57BL/6*^Nlrp1b(129S1)^* transgenic mice, consistent with retention of one dominant LT-responsive allele of *Nlrp1b*, and further suggesting that both CAST/Ei and 129S1 alleles do in fact function in a similar, dominant manner to drive a less severe ERP. Therefore, we propose a second model, wherein at least one additional gene in the critical region of chromosome 11 is required for full expressivity of the ERP and high-level resistance to spore challenge. While we cannot exclude a novel role in inflammation and/or mechanism of action for the CAST/Ei allele of *Nlrp1b,* the findings presented here are more consistent with contributions by an additional gene. Of note, this model does not rule out the possibility that multiple or different genes contribute to the host response to MDP + LPS, LT, and/or spores. Indeed, the critical region contains numerous genes with established or proposed roles in inflammation (**Table S1**).

Three murine QTL, *Lethal toxin sensitivity 1-3* (*Ltxs1-3*), and one gene, *Nlrp1b* (within *Ltxs1*), have been reported to control macrophage and/or whole animal sensitivity to LT [14], [46], [47]. Although backcross mapping data presented here eliminates a role for *Ltxs3* contributing to the ERP, it is possible that one or more genes within *Ltxs1* and/or *2* contribute to the severity of this phenotype. Notably, while the gene at *Ltxs1* responsible for macrophage pyroptosis was identified as *Nlrp1b* [14], a dominantly inherited resistance to LT was also mapped to this region [46], indicating that more than one overlapping QTL may exist at *Ltxs1*. Indeed, in contrast to LT-resistance phenotypes reported for *Ltxs1* and *Ltxs2* [46], allelic variation in B6.CAST.11 animals does not alter susceptibility to LT, defined as time to moribund behavior, compared to B6 mice (**Figure 1C**). Therefore, we propose that additional QTL influencing host response to LT reside within this critical region.

In order to identify candidate genes within the critical region that potentially contribute to ERP and resistance to *B. anthracis* Sterne, we mined a gene expression dataset from a B6 x CAST/EiJ F_2_ cross for cis-acting eQTL. Variation in basal transcription levels of genes within a disease QTL has been used previously to identify candidate genes controlling phenotypic variation [42], [48]. Given the rapid response to LT, as quickly as 18 minutes post i.v. challenge, we reasoned that alterations in basal gene expression levels and/or protein activity could account for altered ERP strength between B6.CAST.11M and C57BL/6*^Nlrp1b(129S1)^* transgenic mice. We further refined the selection criteria for candidate genes to include the presence of single nucleotide polymorphisms within the coding region and known association of candidate genes with inflammatory responses. Finally, we considered other genes previously identified as candidates for host response to LT. The gene encoding inducible nitric oxide synthase (*iNOS/NOS2*) was previously studied with respect to host response to lethal toxin and was identified as overlapping with *Ltxs3* [24], [36], [46], [49]. However, *Nos2* falls outside the critical region reported here and was therefore excluded. Within the current critical region, one gene, *Mgl1/Clec10a,* and one QTL controlling susceptibility to Salmonella infection, *Ity2*, were proposed as candidates for controlling host response at *Ltxs2* [46]. *Mgl1/Clec10a*, and the neighboring *Mgl2,* were identified here as high-priority candidates as they encode galactose/N-acetyl-galactosamine binding lectins expressed on alternatively activated macrophages [50]. Alternatively activated macrophages modulate inflammatory responses and *Mgl1* ^−/−^ mice displayed more severe inflammation in a model of experimental colitis [51].

From the list of high priority eQTL candidates, it is noteworthy that several play a role in inflammatory responses by acting through lipid mediators. *Alox8, Alox12e* and *Alox15* belong to a family of arachidonate lipoxygenases responsible for production of anti-inflammatory lipoxins from arachidonic acid. Lipoxins are predicted to suppress vascular changes induced by inflammatory mediators [52]. Similarly, proteins encoded by phospholipase D2, *Pld2,* phospholipid scramblase 3, *Plscr3,* phosphoinositide-3-kinase, regulatory subunit 6 *Pik3r6,* and spinster homolog 2, *Spns2*, are involved in phospholipid synthesis and/or signaling events associated with inflammatory responses including leukocyte migration, phagocytosis, oxidative burst, and vascular permeability.

β-arrestin 2 (*Arrb2*) and netrin 1 (*Ntn1*) are two additional high priority candidates, and are involved in signaling in response to inflammatory stimuli [53]–[56]. Indeed, *Arrb2* regulates LPS-induced inflammatory response and endotoxemia [54], [55], while *Ntn1* can minimize inflammatory damage associated with ischemia-reperfusion injury [56]. Additional eQTLs were identified in mediators of host innate immunity, including complement C1q binding protein, C1qBP, and the chemokine CXCL16. CXCL16 is elevated in patients with inflammatory bowel disease, and *Cxcl16* ^−/−^ mice display less inflammation in a murine model of enterocolitis [57]. Platelet-activating factor acetylhydrolase, isoform 1b, subunit 1 (*Pafah1b1*), another high-priority candidate, was implicated in susceptibility to necrotizing enterocolitis in humans, and *Pafah1b1* deficiency in mice led to increased susceptibility to this disease [58]. Finally, two eQTLs were identified that have opposite functions in protein metabolism. Eukaryotic initiation factor 4A-I, *Eif4a1*, is a component of the ribosome involved in protein translation, while *Psmb6* (proteasome (prosome, macropain) subunit, beta type 6) is a component of the proteasome involved in protein degradation. Interestingly, PSMB6 is replaced by an alternative proteasome subunit, LMP2, to form an “immunoproteasome” in response to interferon signaling, and has been identified as a candidate gene contributing to autoimmune type-1 diabetes in mice [59].

While differential gene expression has been used extensively with positional cloning efforts to identify candidate genes [42], [59], [60], it is possible that genes responsible for ERP and resistance to *B. anthracis* Sterne infection are not differentially regulated at the level of transcription. For example, the critical region encodes two paralogues of *Nlrp1b, Nlrp1a and Nlrp1c,* that were not identified as cis-acting eQTL. Very little is known regarding these paralogues, and it is possible that they have a role in the increased severity of ERP seen in B6.CAST.11 animals.

NLRP1B inflammasome formation allows for the processing and extracellular release of proinflammatory cytokines. The LT-induced ERP is coupled with the induction of several cytokines, of which IL-1β is one of the earliest detectable in the sera of intoxicated mice (**Figure 2**). IL-1β activity was not required for the ERP, as LT-challenged B6*^Nlrp1b^* ^(129S1);IL-1R−/−^ animals showed ataxia and hypothermic responses that were indistinguishable from B6*^Nlrp1b^* ^(129S1)^ mice (**Figure 3C**). Interestingly, B6.CAST.11M, but not B6 mice challenged i.p. with recombinant IL-1β displayed ataxia (J.K.T. and S.M.L., unpublished observation), suggesting that this cytokine is sufficient but not necessary to bestow the ERP. In contrast, IL-1R deficient B6*^Nlrp1b^* ^(129S1)^ mice were equally susceptible to infection compared to IL-1R deficient mice expressing only B6 alleles of *Nlrp1b*, demonstrating that this cytokine is required for *Nlrp1b*-mediated resistance to *B. anthracis*. In total, these data indicate that IL-1β plays different roles, i.e., for the ERP it is sufficient but not necessary, but for resistance to anthrax it is necessary. One interpretation is that NLRP1B-mediated pyroptosis results in release of IL-1β that can induce multiple other inflammatory cytokines and/or inflammatory mediators that contribute, in a redundant manner, to ataxia, hypothermia, and other ERP-associated clinical signs, but that only a few are critical for resistance to *B. anthracis*. Indeed, we did not observe TNFα release from LT-treated BMDMs (**Figure** **1F**), indicating that other cell types may be responsible for the TNFα response, and possibly hypothermia, in LT-treated mice (**Figures 1B** **,** **2**). This may occur either directly, in response to LT, or indirectly, in response to IL-1β. Conversely, TNFα, but not IL-1β was released from BMDMs derived from all three mouse strains in response to MDP/LPS under the conditions tested (**Figure 1F**), consistent with a *Nlrp1b-*independent role for TNFα in LPS-induced hypothermia. Whether the exacerbated response of B6.CAST.11M mice to LT and MDP/LPS derive from a common mediator, or whether these congenic mice are more responsive to multiple mediators is not currently known. The identities and mechanisms of action of such mediators are currently under investigation.

Our understanding of the role of cytokines in response to LT challenge and *B. anthracis* infection has recently changed. Studies presented here and [15] indicate that cytokines are not a major influence of mortality in response to purified LT injection as once thought, and we find no evidence of alterations in time-to-death as a result of *Nlrp1b* allele status. In contrast, a modest influence of LT-responsive alleles of *Nlrp1b* has been reported in a different murine intoxication model [17], [24]. Regardless of the role of cytokines in response to LT challenge, it is clear that IL-1β is critical for resistance during infection with *B. anthracis* Sterne [17], [36], [37], [61]. Importantly, we extend these prior findings and demonstrate here for the first time that IL-1β is required for protection from infection bestowed by LT-responsive alleles of Nlrp1b.

Septic shock and autoinflammatory diseases such as vitiligo, Crohn's disease, Muckle-Wells syndrome, and gout, among others, result from overactive release of proinflammatory cytokines including IL-1β [62]. Based on differential inflammatory response to disparate stimuli such as LT and LPS/MDP, we propose that B6.CAST.11 and B6*^Nlrp1b(129S1)^* mice may provide a unique system to further analyze inflammatory syndromes. Systemic Inflammatory Response Syndrome (SIRS) is a term used for the generalized inflammatory reaction that occurs in patients undergoing sepsis or a non-septic trauma, such as pancreatitis, hemorrhagic shock, thermal injury, or severe surgery [63]. Accordingly, SIRS is defined by the presentation of two or more of the following clinical markers that match LT-induced ERP: a body temperature higher than 38°C or lower than 36°C, endothelial dysfunction and increased microvascular permeability, and platelet sludging causing maldistribution of blood flow [64], [65]. A second inflammatory syndrome, Compensatory Anti-inflammatory Response Syndrome (CARS), was more recently defined as a counter-regulatory response to the overzealous inflammatory reaction that occurs in SIRS [29]. This adaptive response is characterized by the increased induction of anti-inflammatory molecules IL-10 and IL-4 [29]. Although attenuation of inflammation may be beneficial in some instances, CARS patients are often more susceptible to secondary bacterial infections [29], [66]. Interestingly, B6*^Nlrp1b^* ^(129S1)^ transgenic mice display a weaker ERP (**Figure 1**), are more susceptible to *B. anthracis* infection (**Figure 4**), and release higher levels of IL-4 than B6.CAST.11M mice in response to LT (**Figure 2**). Because the gene encoding IL-4 resides at 53.4 Mb on chromosome 11 (part of the original critical region), we tested the role of this cytokine in the differential response to LT. B6*^Nlrp1b^* ^(129S1)*;IL4*−/−^ mice showed no alteration in the ERP or resistance to *B. anthracis* compared to B6*^Nlrp1b^* ^(129S1)^ animals, indicating this cytokine cannot, on its own, account for differences in hypothermia, ataxia, or resistance to *B. anthracis* between B6*^Nlrp1b^* ^(129S1)^ and B6.CAST.11M animals. However, mice lacking both an LT-responsive allele of *Nlrp1b* and the *IL4* gene were slightly more resistant to *B. anthracis* spores compared to B6 animals (**Figure 4F**), suggesting a negative role for Th_2_ responses in the outcome of *B. anthracis* infection. Further mapping confirmed that *IL4* lies outside the current critical region (**Figure 6**), eliminating it as a candidate involved in the regulation of the ERP. Notably, IL-10 levels were equivalent between B6*^Nlrp1b(129S1)^* and B6.CAST.11M mice following LT challenge (**Figure 2**). Therefore, we hypothesize allelic variation of gene(s) in the chromosome 11 critical region represent a novel genetic control mechanism for a SIRS/CARS like inflammatory response to LT. B6.CAST.11M mice further showed increased responsiveness to a MDP + LPS model of sepsis compared with B6 controls (**Figure 1E**), indicating that gene(s) within the critical region may play a role in multiple inflammatory syndromes including sepsis. Currently, no single treatment for sepsis exists [67], and there is little understanding of mechanisms driving resolution of shock/sepsis [68], [69]. We predict that identification of genetic and molecular mechanisms controlling severity of LT-induced ERP and/or host response to MDP + LPS will provide insight into novel intervention strategies for sepsis and other inflammatory diseases.

## Materials and Methods

### Animal Maintenance and Breeding

All studies involving the use of mice were conducted in compliance with the Animal Welfare Act and other federal statutes and regulations relating to animals and experiments involving animals and adheres to principles stated in the Guide for the Care and Use of Laboratory Animals, National Research Council, 1996. All studies involving the use of mice were approved by the University of California Animal Research Committee and/or the USAMRIID Animal Care and Use Committee (Permit numbers: 2005-122 and 2007-019). UCLA and USAMRIID are fully accredited by the Association for the Assessment and Accreditation of Laboratory Animal Care International.

B6 and CAST/EiJ mice were purchased from the Jackson laboratory (Bar Harbor, ME). Transgenic mice expressing a 129S1/SvImJ (129S1)-derived LT^S^ allele of *Nlrp1b/Nalp1b* on a LT-resistant (LT^R^) B6 background (B6*^Nlrp1b^* ^(129S1)^) and backcrossed to B6 for seven generations were obtained from Drs. E. Boyden and W. Dietrich (Harvard Medical School) [14], [15]. B6*^Nlrp1b^* ^(129S1)^ mice both heterozygous and homozygous for the *Nlrp1b* transgene were used for experiments reported here as no difference in response to LT or Sterne spores was observed. The library of congenic mice consisting of introgressed segments of CAST/Ei DNA on a B6 background (B6.CAST) has been described [25]. B6.CAST.11M mice were crossed to B6 to obtain [B6.CAST.11M x B6] F_1_ offspring used for intoxication experiments and for determining the mode of inheritance. Backcross progeny were generated by crossing B6.CAST.11M mice to B6 mice, and backcrossing the [B6.CAST.11M x B6] F_1_ mice to B6.CAST.11M mice.

### Mouse Genotyping

Genomic DNA was isolated from tail biopsies using Qiagen DNeasy blood and tissue kit. Presence of *Nlrp1b*(129S1) transgene was monitored as previously described [15]. Mutant and wild type alleles of *Il1r1* were identified using PCR primers oIMR000160, oIMR0161, oIMR7898, and oIMR7899 as per supplier's protocol (Jackson Laboratory, Bar Harbor, ME). Mutant and wild type alleles of *Il4* were identified using PCR primers oIMR0077, oIMR0078, and oIMR0079 (Jackson Laboratory, Bar Harbor, ME). [B6.CAST.11M x B6] F_1_ x B6.CAST.11M backcross progeny were genotyped at multiple positions within the region of interest using chromosome 11 microsatellite markers that distinguish CAST/Ei and B6 alleles (Mouse Genome Informatics (MGI) database (http://www.informatics.jax.org)). All 139 backcross progeny were genotyped at D11MIT190 (47.6 Mbp), D11MIT131 (55.9 Mpb), D11MIT260 (61.6 Mbp), D11Die36 (70.9 Mbp), D11MIT357 (90.1 Mbp), and D11MIT199 (101.7 Mbp) by PCR and gel electrophoresis. Backcross progeny that displayed genetic recombination within this region and/or presented with a strong ERP were further genotyped at the polymorphic microsatellite markers indicated in **Figure 6B**.

### Toxin Preparation

LT components were expressed and purified as previously described [15]. Specifically, the PA expression plasmid, PA-pET22b was provided by Dr. John Collier (Harvard Medical School) and transformed into *Escherichia coli* BL21 DE3 cells. A fresh colony was inoculated into a 20 mL starter culture of Luria Bertani (LB) Lennox media (EMD Biosciences, Inc.) with 100 mg/mL ampicillin and grown overnight at 37°C. The following day a 1∶50 dilution was made into a 2 L baffled Erlenmeyer flask of LB Lennox supplemented with 100 mg/mL ampicillin. The culture was grown at 37°C and shaking at 250 rpm until an optical density of 1.0 was reached. The culture was then induced with a final concentration of 1 mM isopropyl β-D-1-thiogalactopyranoside and allowed to grow at 30°C and shaking at 250 rpm for 4 h. PA was isolated from the periplasm and purified over a Macro-Prep HighQ (BioRad) column. LF expressed and purified from *B. megaterium* was obtained from Dr. Jeremy Mogridge (University of Toronto), and resuspended in pharmaceutical grade saline for all animal experiments. A dose of 5 µg PA and 2.5 µg LF per g body weight was diluted in pharmaceutical grade saline and injected i.p. Alternatively, PA and LF were purified from *B. anthracis* strain BH450 as described [70]. LF produced from strain BH450 displayed 3-fold lower activity [71], and consequently a dose of 15 µg PA and 7.5 µg LF per g body weight was used to achieve a similar mortality rate [15]. Endotoxin was removed from PA and LF protein preparations using the Detoxi-Gel Endotoxin Removing Gel (Pierce). Purified proteins were assayed for endotoxin using the Limulus Amebocyte Lysate kit (BioWhittaker/Lonza Bioscientific), which detects a minimum of 0.03 endotoxin units/mL.

### Lethal Toxin Challenge

A total of 36 B6.CAST congenic strains were screened for LT response. For each strain, ∼5 mice of mixed gender and ∼8 weeks of age were administered LT by i.p. injection at a dose that induced mortality in B6 control animals with a mean time to death of ∼60 h (see above). Larger-scale secondary screens were performed using 5–10 additional animals of each candidate or control strain. Upon injection, mice were closely monitored for ∼2 h and then every 2–3 h for clinical signs consisting of ataxia, bloat, lethargy, loose feces and/or hunched posture. Ataxia was measured using a grading system in which mice were scored as displaying a mild, moderate, or severe phenotype. A mild phenotype was defined as reduced exploratory behavior or rearing on hindlimbs, a slower and/or less steady gait, but free ambulation throughout the cage. A moderate score was defined as a preferred sedentary state, but the mouse was able to generate a slow, unsteady (e.g. wobbly) gait for up to ∼7 sec before resting. A severe score was defined as a stationary state, but upon stimulation the mouse could generate a few unstable steps (e.g. severe wobble and/or tremor) before stopping.

For experiments focused on the acute presentation of clinical signs, mice were closely monitored for ∼6–8 hours then euthanized. Mouse body temperatures were measured following LT injection using a rectal thermometer probe. Baseline temperatures were determined prior to LT injection and no differences were observed between animal groups (not shown). Temperatures were recorded hourly for up to 7 h following LT injection. Some mice were injected i.p. with recombinant murine IL-1β (R & D Systems) at a dose of 100 ng per mouse immediately prior to LT injection and observed as described above.

*In vitro* BMDM intoxication studies were performed as previously described [72]. Briefly, bone marrow was flushed from femur and tibia bones of 8-week old mice using DMEM supplemented with 10% fetal bovine serum (FBS) and 1% penicillin-streptomycin-glutamine (PSG) cocktail (Invitrogen). Marrow was then briefly centrifuged at 1500 rpm and resuspended in 1x ACK Lysis Buffer (150 mM NH_4_Cl, 1 mM KHCO_3_, 100 µM Na_2_-EDTA, pH 7.2) for 5 min. Cells were briefly centrifuged at 1500 rpm and resuspended in DMEM supplemented with 10% FBS, PSG, and 2% conditioned medium of CMG14-12 cells as a source of M-CSF. Ten million cells were seeded per 15 cm plate and incubated at 37°C, 5% CO_2_ for 6–7 days, then harvested and seeded at 5×10^4^ cells per well in a 96 well plate in DMEM supplemented with 10% FBS, PS cocktail (Invitrogen), 25 mM HEPES, and Glutamax (Invitrogen). PA and LF were titrated in a final volume of 100 µL / well and incubated for 4 h prior to addition of ATPlite 1-step reagent (PerkinElmer, Waltham, Massachusetts). Luminescence intensity was measured using Victor 3V (PerkinElmer) plate reader. Alternatively, BMDMs were exposed to 0.1 µg/mL MDP and 0.1 ng/mL LPS for 8 hr [27], or 250 ng/mL PA and 250 ng/mL LF for 3 hr prior to collecting cell culture supernatant for cytokine analysis.

### Cytokine Analysis

For cytokine analysis, mice were injected i.p. with LT at a dose of 15 µg PA and 7.5 µg LF per g body weight. Blood was collected via cardiac puncture and allowed to coagulate for ∼1 h. Samples were centrifuged, and sera was collected and stored at −80°C. Cytokines were detected using the Millipore Milliplex MAP Mouse Cytokine Kit per manufacturer's instructions and read on a Luminex 100 IS or BioRad Bioplex 200 instrument. Serum from each mouse was analyzed in duplicate and average values from these independent measurements were used to calculate a mean for each animal group (n = 5). For BMDM experiments, at least two independent mice per strain were used, and cells from each mouse were tested in duplicate. All cytokine data were analyzed using MILLIPLEX Analyst software (EMD-Millipore) with five parameter logistic curve fitting.

### Spore Challenge and Cellular Analysis Experiments

B6.CAST.11M, B6*^Nlrp1b^* ^(129S1)^, and non-transgenic littermate/cagemate (i.e. B6) mice were challenged with unencapsulated, toxigenic Sterne strain (7702) by i.p. injection as indicated in figure legends and monitored daily for 14 days. B6.CAST.11M, B6*^Nlrp1b^* ^(129S1)^, and B6 mice were also used for peritoneal cellular infiltration determination following spore challenge. For cellular analysis, mice were infected i.p. with ∼1.6×10^7^ Sterne spores and euthanized at 4, 28, 52, 76, and 135 h post infection. Uninfected mice were used to determine baseline cell populations in the peritoneal cavity of each strain (t = 0). Peritoneal exudates from infected mice were harvested by injecting 7 ml sterile HBSS and 3 ml air into the peritoneal cavity. The fluid was agitated within the cavity and then extracted. The fluids were analyzed as follows from three mice per group at each time point, except where indicated. Total cell counts were determined microscopically by using a hemocytometer; four fields for each mouse were counted and averaged. The cells from an aliquot of sample were then collected onto slides with a Cytospin centrifuge (Shandon, Inc., Pittsburgh, PA). The slides were fixed in methanol, stained with Diff-Quik (Harleco, Philadelphia, PA), and then evaluated microscopically to determine the percentages of cell types (i.e., percentages of monocytes, PMN). The average % of each cell type per mouse group was calculated, and the total number of each cell type was determined by multiplying the mean proportion of each cell type by the mean hemocytometer count for each mouse group. These values were plotted including standard deviation for each mouse group.

### Expression QTL Analysis

Comprehensive mapping of gene expression in adipose, brain, liver and muscle of 442 F2 progeny of a cross between C57BL/6J and CAST/Ei mice was previously described [73]. From this dataset, we selected genes with strong (LOD >4.3) cis-acting expression-QTLs located in the region between 66.36 and 74.67 on Chromosome 11. Peak LOD scores for each tissue are reported on **Table S1**. Gene names, positions, and functions were compared to NCBI build 37.1 and MGI annotation datasets. Genes were prioritized by biological function as determined by MGI genome analysis tools (www.informatics.jax.org/tools.shtml) combined with manual curation (www.ncbi.nlm.nih.gov/gene). B6 and CAST/Ei nucleotide sequences were compared using fully sequenced genomes from each strain available at http://www.sanger.ac.uk/cgi-bin/modelorgs/mousegenomes/snps.pl to identify single nucleotide polymorphisms (SNPs). Consistent with genetic divergence between these strains, 64,102 SNPs were identified between 63.36–74.67 Mbp. The search was refined to analyze only predicted mRNA sequences, resulting in 2,421 SNPs that mapped to 74 out of the 81 eQTL.

## Supporting Information

Table S1 **Candidate genes within the critical region on chromosome 11.** Dataset analysis of over 1,600 microarray experiments performed on 442 F_2_ (B6 x CAST/EiJ) mice revealed 81 *cis*-acting eQTL (differences in gene expression map to structural changes in genes themselves). Of the 81, 25 (shown in bold) were identified as high priority candidates based on their known or predicted functions in inflammation and/or immunological processes. In addition, the critical region encodes *Nlrp1a,* and *Nlrp1c*, which were not analyzed by microarray, but which are potential candidates for controlling ERP and resistance to *B. anthracis* Sterne.
(PDF)

## References

[1] Brodsky IE, Medzhitov R (2009). Targeting of immune signalling networks by bacterial pathogens.. Nat Cell Biol.

[2] Pitzschke A, Schikora A, Hirt H (2009). MAPK cascade signalling networks in plant defence.. Curr Opin Plant Biol.

[3] Banks DJ, Ward SC, Bradley KA (2006). New insights into the functions of anthrax toxin.. Expert Rev Mol Med.

[4] Dang O, Navarro L, Anderson K, David M (2004). Cutting edge: anthrax lethal toxin inhibits activation of IFN-regulatory factor 3 by lipopolysaccharide.. J Immunol.

[5] Erwin JL, DaSilva LM, Bavari S, Little SF, Friedlander AM (2001). Macrophage-derived cell lines do not express proinflammatory cytokines after exposure to Bacillus anthracis lethal toxin.. Infect Immun.

[6] Hsu LC, Park JM, Zhang K, Luo JL, Maeda S (2004). The protein kinase PKR is required for macrophage apoptosis after activation of Toll-like receptor 4.. Nature.

[7] Park JM, Greten FR, Li ZW, Karin M (2002). Macrophage apoptosis by anthrax lethal factor through p38 MAP kinase inhibition.. Science.

[8] Popov SG, Villasmil R, Bernardi J, Grene E, Cardwell J (2002). Effect of Bacillus anthracis lethal toxin on human peripheral blood mononuclear cells.. FEBS Lett.

[9] Hsu LC, Ali SR, McGillivray S, Tseng PH, Mariathasan S (2008). A NOD2-NALP1 complex mediates caspase-1-dependent IL-1beta secretion in response to Bacillus anthracis infection and muramyl dipeptide.. Proc Natl Acad Sci U S A.

[10] Cui X, Li Y, Li X, Haley M, Moayeri M (2006). Sublethal doses of Bacillus anthracis lethal toxin inhibit inflammation with lipopolysaccharide and Escherichia coli challenge but have opposite effects on survival.. J Infect Dis.

[11] Bergman NH, Passalacqua KD, Gaspard R, Shetron-Rama LM, Quackenbush J (2005). Murine macrophage transcriptional responses to Bacillus anthracis infection and intoxication.. Infect Immun.

[12] Liu S, Miller-Randolph S, Crown D, Moayeri M, Sastalla I (2010). Anthrax toxin targeting of myeloid cells through the CMG2 receptor is essential for establishment of *Bacillus anthracis* infections in mice.. Cell Host Microbe.

[13] Cote CK, Dimezzo TL, Banks DJ, France B, Bradley KA (2008). Early interactions between fully virulent *Bacillus anthracis* and macrophages that influence the balance between spore clearance and development of a lethal infection.. Microbes Infect.

[14] Boyden ED, Dietrich WF (2006). Nalp1b controls mouse macrophage susceptibility to anthrax lethal toxin.. Nat Genet.

[15] Terra JK, Cote CK, France B, Jenkins AL, Bozue JA (2010). Cutting edge: resistance to Bacillus anthracis infection mediated by a lethal toxin sensitive allele of Nalp1b/Nlrp1b.. J Immunol.

[16] Nye SH, Wittenburg AL, Evans DL, O'Connor JA, Roman RJ (2007). Rat survival to anthrax lethal toxin is likely controlled by a single gene.. Pharmacogenomics J.

[17] Moayeri M, Crown D, Newman ZL, Okugawa S, Eckhaus M (2010). Inflammasome sensor Nlrp1b-dependent resistance to anthrax is mediated by caspase-1, IL-1 signaling and neutrophil recruitment.. PLoS Pathog.

[18] Lincoln RE, Walker JS, Klein F, Rosenwald AJ, Jones WI (1967). Value of field data for extrapolation in anthrax.. Fed Proc.

[19] Welkos SL, Keener TJ, Gibbs PH (1986). Differences in susceptibility of inbred mice to *Bacillus anthracis*.. Infect Immun.

[20] Welkos SL, Friedlander AM (1988). Pathogenesis and genetic control of resistance to the Sterne strain of *Bacillus anthracis*.. Microb Pathog.

[21] Welkos SL, Trotter RW, Becker DM, Nelson GO (1989). Resistance to the Sterne strain of *B. anthracis*: phagocytic cell responses of resistant and susceptible mice.. Microb Pathog.

[22] Welkos SL, Vietri NJ, Gibbs PH (1993). Non-toxigenic derivatives of the Ames strain of Bacillus anthracis are fully virulent for mice: role of plasmid pX02 and chromosome in strain-dependent virulence.. Microb Pathog.

[23] Harvill ET, Lee G, Grippe VK, Merkel TJ (2005). Complement depletion renders C57BL/6 mice sensitive to the Bacillus anthracis Sterne strain.. Infect Immun.

[24] Moayeri M, Martinez NW, Wiggins J, Young HA, Leppla SH (2004). Mouse susceptibility to anthrax lethal toxin is influenced by genetic factors in addition to those controlling macrophage sensitivity.. Infect Immun.

[25] Davis RC, Jin A, Rosales M, Yu S, Xia X (2007). A genome-wide set of congenic mouse strains derived from CAST/Ei on a C57BL/6 background.. Genomics.

[26] Roberts JE, Watters JW, Ballard JD, Dietrich WF (1998). Ltx1, a mouse locus that influences the susceptibility of macrophages to cytolysis caused by intoxication with Bacillus anthracis lethal factor, maps to chromosome 11.. Mol Microbiol.

[27] Shikama Y, Kuroishi T, Nagai Y, Iwakura Y, Shimauchi H (2011). Muramyldipeptide augments the actions of lipopolysaccharide in mice by stimulating macrophages to produce pro-IL-1{beta} and by down-regulation of the suppressor of cytokine signaling 1 (SOCS1).. Innate Immun.

[28] Biedermann T, Rocken M (2005). Pro- and anti-inflammatory effects of IL-4: from studies in mice to therapy of autoimmune diseases in humans.. Ernst Schering Res Found Workshop.

[29] Adib-Conquy M, Cavaillon JM (2009). Compensatory anti-inflammatory response syndrome.. Thromb Haemost.

[30] Jones JW, Kayagaki N, Broz P, Henry T, Newton K (2010). Absent in melanoma 2 is required for innate immune recognition of Francisella tularensis.. Proc Natl Acad Sci U S A.

[31] Hart PH, Vitti GF, Burgess DR, Whitty GA, Piccoli DS (1989). Potential antiinflammatory effects of interleukin 4: suppression of human monocyte tumor necrosis factor alpha, interleukin 1, and prostaglandin E2.. Proc Natl Acad Sci U S A.

[32] Vannier E, Miller LC, Dinarello CA (1992). Coordinated antiinflammatory effects of interleukin 4: interleukin 4 suppresses interleukin 1 production but up-regulates gene expression and synthesis of interleukin 1 receptor antagonist.. Proc Natl Acad Sci U S A.

[33] Cui X, Moayeri M, Li Y, Li X, Haley M (2004). Lethality during continuous anthrax lethal toxin infusion is associated with circulatory shock but not inflammatory cytokine or nitric oxide release in rats.. Am J Physiol Regul Integr Comp Physiol.

[34] Tournier JN, Quesnel-Hellmann A, Mathieu J, Montecucco C, Tang WJ (2005). Anthrax edema toxin cooperates with lethal toxin to impair cytokine secretion during infection of dendritic cells.. J Immunol.

[35] Hughes MA, Green CS, Lowchyj L, Lee GM, Grippe VK (2005). MyD88-dependent signaling contributes to protection following Bacillus anthracis spore challenge of mice: implications for Toll-like receptor signaling.. Infect Immun.

[36] Kalns J, Scruggs J, Millenbaugh N, Vivekananda J, Shealy D (2002). TNF receptor 1, IL-1 receptor, and iNOS genetic knockout mice are not protected from anthrax infection.. Biochem Biophys Res Commun.

[37] Kang TJ, Basu S, Zhang L, Thomas KE, Vogel SN (2008). *Bacillus anthracis* spores and lethal toxin induce IL-1beta via functionally distinct signaling pathways.. Eur J Immunol.

[38] Okugawa S, Moayeri M, Eckhaus MA, Crown D, Miller-Randolph S (2011). MyD88-dependent signaling protects against anthrax lethal toxin-induced impairment of intestinal barrier function.. Infect Immun.

[39] Henry T, Monack DM (2007). Activation of the inflammasome upon Francisella tularensis infection: interplay of innate immune pathways and virulence factors.. Cell Microbiol.

[40] Doss S, Schadt EE, Drake TA, Lusis AJ (2005). Cis-acting expression quantitative trait loci in mice.. Genome Res.

[41] Schadt EE, Lamb J, Yang X, Zhu J, Edwards S (2005). An integrative genomics approach to infer causal associations between gene expression and disease.. Nat Genet.

[42] Schadt EE, Monks SA, Drake TA, Lusis AJ, Che N (2003). Genetics of gene expression surveyed in maize, mouse and man.. Nature.

[43] Lan H, Rabaglia ME, Stoehr JP, Nadler ST, Schueler KL (2003). Gene expression profiles of nondiabetic and diabetic obese mice suggest a role of hepatic lipogenic capacity in diabetes susceptibility.. Diabetes.

[44] Jansen RC, Nap JP (2001). Genetical genomics: the added value from segregation.. Trends Genet.

[45] Jansen RC (2003). Studying complex biological systems using multifactorial perturbation.. Nat Rev Genet.

[46] McAllister RD, Singh Y, Du Bois WD, Potter M, Boehm T (2003). Susceptibility to anthrax lethal toxin is controlled by three linked quantitative trait Loci.. Am J Pathol.

[47] Watters JW, Dietrich WF (2001). Genetic, physical, and transcript map of the Ltxs1 region of mouse chromosome 11.. Genomics.

[48] Hubner N, Wallace CA, Zimdahl H, Petretto E, Schulz H (2005). Integrated transcriptional profiling and linkage analysis for identification of genes underlying disease.. Nat Genet.

[49] Sebastiani G, Olien L, Gauthier S, Skamene E, Morgan K (1998). Mapping of genetic modulators of natural resistance to infection with Salmonella typhimurium in wild-derived mice.. Genomics.

[50] Raes G, Brys L, Dahal BK, Brandt J, Grooten J (2005). Macrophage galactose-type C-type lectins as novel markers for alternatively activated macrophages elicited by parasitic infections and allergic airway inflammation.. J Leukoc Biol.

[51] Saba K, Denda-Nagai K, Irimura T (2009). A C-type lectin MGL1/CD301a plays an anti-inflammatory role in murine experimental colitis.. A J Pathol.

[52] Godson C, Mitchell S, Harvey K, Petasis NA, Hogg N (2000). Cutting edge: lipoxins rapidly stimulate nonphlogistic phagocytosis of apoptotic neutrophils by monocyte-derived macrophages.. J Immunol.

[53] Wang Y, Tang Y, Teng L, Wu Y, Zhao X (2006). Association of beta-arrestin and TRAF6 negatively regulates Toll-like receptor-interleukin 1 receptor signaling.. Nat Immunol.

[54] Fan H, Bitto A, Zingarelli B, Luttrell LM, Borg K (2010). Beta-arrestin 2 negatively regulates sepsis-induced inflammation.. Immunology.

[55] Porter KJ, Gonipeta B, Parvataneni S, Appledorn DM, Patial S (2010). Regulation of lipopolysaccharide-induced inflammatory response and endotoxemia by beta-arrestins.. J Cell Physiol.

[56] Tadagavadi RK, Wang W, Ramesh G (2010). Netrin-1 regulates Th1/Th2/Th17 cytokine production and inflammation through UNC5B receptor and protects kidney against ischemia-reperfusion injury.. J Immunol.

[57] Uza N, Nakase H, Yamamoto S, Yoshino T, Takeda Y (2011). SR-PSOX/CXCL16 plays a critical role in the progression of colonic inflammation.. Gut.

[58] Lu J, Pierce M, Franklin A, Jilling T, Stafforini DM (2010). Dual roles of endogenous platelet-activating factor acetylhydrolase in a murine model of necrotizing enterocolitis.. Pediatr Res.

[59] Ivakine EA, Gulban OM, Mortin-Toth SM, Wankiewicz E, Scott C (2006). Molecular genetic analysis of the Idd4 locus implicates the IFN response in type 1 diabetes susceptibility in nonobese diabetic mice.. J Immunol.

[60] Sancho-Shimizu V, Khan R, Mostowy S, Lariviere L, Wilkinson R (2007). Molecular genetic analysis of two loci (Ity2 and Ity3) involved in the host response to infection with Salmonella typhimurium using congenic mice and expression profiling.. Genetics.

[61] Ali SR, Timmer AM, Bilgrami S, Park EJ, Eckmann L (2011). Anthrax toxin induces macrophage death by p38 MAPK inhibition but leads to inflammasome activation via ATP leakage.. Immunity.

[62] Franchi L, Eigenbrod T, Munoz-Planillo R, Nunez G (2009). The inflammasome: a caspase-1-activation platform that regulates immune responses and disease pathogenesis.. Nat Immunol.

[63] Castellheim A, Brekke OL, Espevik T, Harboe M, Mollnes TE (2009). Innate immune responses to danger signals in systemic inflammatory response syndrome and sepsis.. Scand J Immunol.

[64] Takahashi H, Tsuda Y, Kobayashi M, Herndon DN, Suzuki F (2006). CCL2 as a trigger of manifestations of compensatory anti-inflammatory response syndrome in mice with severe systemic inflammatory response syndrome.. J Leukoc Biol.

[65] Klempner MS, Talbot EA, Lee SI, Zaki S, Ferraro MJ (2010). Case records of the Massachusetts General Hospital. Case 25-2010. A 24-year-old woman with abdominal pain and shock.. N Engl J Med.

[66] Torre D, Tambini R, Aristodemo S, Gavazzeni G, Goglio A (2000). Anti-inflammatory response of IL-4, IL-10 and TGF-beta in patients with systemic inflammatory response syndrome.. Mediators Inflamm.

[67] Peyssonnaux C, Cejudo-Martin P, Doedens A, Zinkernagel AS, Johnson RS (2007). Cutting edge: Essential role of hypoxia inducible factor-1alpha in development of lipopolysaccharide-induced sepsis.. J Immunol.

[68] Ayala A, Chung CS, Grutkoski PS, Song GY (2003). Mechanisms of immune resolution.. Crit Care Med.

[69] Martinon F (2010). Update on biology: uric acid and the activation of immune and inflammatory cells.. Curr Rheumatol Rep.

[70] Park S, Leppla SH (2000). Optimized production and purification of *Bacillus anthracis* lethal factor.. Protein Expr Purif.

[71] Gupta PK, Moayeri M, Crown D, Fattah RJ, Leppla SH (2008). Role of N-terminal amino acids in the potency of anthrax lethal factor.. PLoS One.

[72] Averette KM, Pratt MR, Yang Y, Bassilian S, Whitelegge JP (2009). Anthrax lethal toxin induced lysosomal membrane permeabilization and cytosolic cathepsin release is Nlrp1b/Nalp1b-dependent.. PLoS ONE.

[73] Schadt EE, Molony C, Chudin E, Hao K, Yang X (2008). Mapping the genetic architecture of gene expression in human liver.. PLoS Biol.

