# Supplementary material for: Allelic Variation on Murine Chromosome 11 Modifies Host Inflammatory Responses and Resistance to Bacillus anthracis
Source: PLoS Pathog. 2011 Dec 29;7(12):e1002469. doi: 10.1371/journal.ppat.1002469 (PMC3248472; doi:10.1371/journal.ppat.1002469)
Supplement: Table S1 — Candidate genes within the critical region on chromosome 11. Dataset analysis of over 1,600 microarray experiments performed on 442 F2 (B6 x CAST/EiJ) mice revealed 81 cis-acting eQTL (differences in gene expression map to structural changes in genes themselves). Of the 81, 25 (shown in bold) were identified as high priority candidates based on their known or predicted functions in inflammation and/or immunological processes. In addition, the critical region encodes Nlrp1a, and Nlrp1c, which were not analyzed by microarray, but which are potential candidates for controlling ERP and resistance to B. anthracis Sterne. (PDF) [file ppat.1002469.s001.pdf]

TABLE S1

Position				Peak eQTL LOD score			
cM	start (base position)	Symbol	Gene Name	adipose	brain	liver	muscle
40.59	66849006	c330020G15Rik	RIKEN cDNA C330020G15 gene	4.79	7.97	13.43	7.40
40.59	66851407	2310004I24Rik	RIKEN cDNA 2310004I24 gene		11.05	13.88	
**40.59**	**66866172**	**Sco1**	**SCO cytochrome oxidase deficient homolog 1 (yeast)**	**7.87**	**8.37**	**45.82**	**50.85**
40.59	66891802	Myh3	myosin, heavy polypeptide 3, skeletal muscle, embryonic	14.21	34.87	18.84	
40.59	66984568	Myh2	myosin, heavy polypeptide 2, skeletal muscle, adult		7.51		
40.59	67090626	Myh8	myosin, heavy polypeptide 8, skeletal muscle, perinatal	8.52	38.23		25.55
41.13	67268939	Gas7	growth arrest specific 7	15.86	45.85	33.12	32.26
**41.13**	**67475004**	**Glp2r**	**glucagon-like peptide 2 receptor**		**57.45**		**4.71**
41.16	67738308	Wdr16	WD repeat domain 16		84.99		85.37
**41.45**	**68022871**	**Ntn1**	**netrin 1**	**15.61**	**5.74**	**13.71**	**13.86**
**41.76**	**68368256**	**Pik3r6**	**phosphoinositide-3-kinase, regulatory subunit 6**	**38.33**	**36.58**		**11.23**
41.81	68369688	Mfsd6l	major facilitator superfamily domain containing 6-like	4.97			
41.95	68505061	Myh10	myosin, heavy polypeptide 10, non-muscle	14.44			
42.09	68634936	Ndel1	nuclear distribution gene E-like homolog 1 (A. nidulans)	25.60		11.62	15.53
**42.21**	**68756659**	**Arhgef15**	**Rho guanine nucleotide exchange factor (GEF) 15**		**25.34**		
**42.24**	**68781633**	**Slc25a35**	**solute carrier family 25, member 35**	**9.21**	**6.56**		
**42.24**	**68785986**	**Rangrf**	**RAN guanine nucleotide release factor**		**20.52**		
42.29	68829413	1500010J02Rik	CST complex subunit CTC1 isoform c			9.00	16.71
42.35	68884308	Tmem107	transmembrane protein 107		64.59		7.05
**42.36**	**68901992**	**Vamp2**	**vesicle-associated membrane protein 2**				**4.65**
**42.38**	**68997387**	**Alox8**	**arachidonate 8-lipoxygenase**		**39.00**		
42.52	69153963	A030009H04Rik	RIKEN cDNA A030009H04 gene		8.58		
42.63	69234311	Dnahc2	dynein, axonemal, heavy chain 2		5.05		
42.86	69413238	Atp1b2	ATPase, Na+/K+ transporting, beta 2 polypeptide				5.16
42.86	69435526	Sat2	spermidine/spermine N1-acetyl transferase 2		43.44	6.60	28.32
42.86	69470199	Mpdu1	mannose-P-dolichol utilization defect 1		17.53	22.37	10.81
**42.86**	**69480438**	**Eif4a1**	**eukaryotic translation initiation factor 4A1**	**15.78**	**42.07**	**30.83**	**24.78**
42.86	69495640	2010012P19Rik	RIKEN cDNA 2010012P19 gene	50.94	49.79	68.15	57.90
42.87	69579414	Zbtb4	zinc finger and BTB domain containing 4	4.70	43.91		
42.87	69597538	Chrnb1	cholinergic receptor, nicotinic, beta polypeptide 1 (muscle)	16.15			36.76
42.89	69636624	Nlgn2	neuroligin 2	15.16		5.62	
42.89	69652016	1810027O10Rik	RIKEN cDNA 1810027O10 gene				6.92
**42.90**	**69660178**	**Plscr3**	**phospholipid scramblase 3**		**13.74**	**11.44**	**10.57**

42.93	69756045	Slc2a4	solute carrier family 2 (facilitated glucose transporter), member 4		63.97		
42.94	69781724	Rai12	retinoic acid induced 12		9.84		
42.99	69906146	Asgr2	asialoglycoprotein receptor 2		24.24		
**42.99**	**69943831**	**Mgl2**	**macrophage galactose N-acetyl-galactosamine specific lectin 2**	**12.60**			**14.41**
**42.99**	**69980125**	**Clec10a**	**C-type lectin domain family 10, member A**		**40.57**		
42.99	70026254	Slc16a11	solute carrier family 16 (monocarboxylic acid transporters), member 11		15.72	11.42	
42.99	70030291	Slc16a13	solute carrier family 16 (monocarboxylic acid transporters), member 13		10.19		
42.99	70048708	0610010K14Rik	RIKEN cDNA 0610010K14 gene				6.00
42.99	70051626	Rnasek	ribonuclease, RNase K	5.49		43.34	31.15
**42.99**	**70129112**	**Alox12e**	**arachidonate lipoxygenase, epidermal**	**16.55**			
**42.99**	**70157654**	**Alox15**	**arachidonate 15-lipoxygenase**	**9.07**			
42.99	70206385	Pelp1	proline, glutamic acid and leucine rich protein 1			8.30	7.55
**42.99**	**70246137**	**Arrb2**	**arrestin, beta 2**	**49.05**	**79.82**	**51.71**	**7.92**
42.99	70265421	Med11	mediator of RNA polymerase II transcription, subunit 11 homolog (S. cerevisiae)	20.86	24.37		21.02
**42.99**	**70267485**	**Cxcl16**	**chemokine (C-X-C motif) ligand 16**				**4.54**
42.99	70272935	Zmynd15	zinc finger, MYND-type containing 15	15.31	16.07	26.75	
42.99	70332698	Gltpd2	glycolipid transfer protein domain containing 2			38.88	
**42.99**	**70338869**	**Psmb6**	**proteasome (prosome, macropain) subunit, beta type 6**	**7.55**	**8.09**	**11.24**	**5.41**
**42.99**	**70353566**	**Pld2**	**phospholipase D2**				**62.75**
43.0	70376392	Mink1	misshapen-like kinase 1 (zebrafish)	5.90		9.07	
43.21	70457698	Slc25a11	solute carrier family 25 (mitochondrial carrier oxoglutarate carrier), member 11				9.12
43.21	70470704	Eno3	enolase 3, beta muscle			9.54	
43.21	70477273	Spag7	sperm associated antigen 7				6.27
43.21	70501863	Inca1	inhibitor of CDK, cyclin A1 interacting protein 1	30.54	25.88	8.35	17.19
43.21	70514050	Kif1c	kinesin family member 1C				58.39
43.21	70577711	Zfp3	zinc finger protein 3			5.24	
43.21	70658280	Rabep1	rabaptin, RAB GTPase binding effector protein 1				4.81
43.21	70756560	Nup88	nucleoporin 88		12.89		11.36
43.21	70783715	Rpain	RPA interacting protein	6.42	54.15		46.55
**43.21**	**70791338**	**C1qbp**	**complement component 1, q subcomponent binding protein**		**18.77**		
43.21	70797593	Dhx33	DEAH (Asp-Glu-Ala-His) box polypeptide 33	33.46	19.19	46.49	12.71
43.21	70820666	Derl2	Der1-like domain family, member 2		14.12		6.03
43.21	70833113	Mis12	MIS12 homolog (yeast)	35.24	18.40	19.58	
43.21	70845451	6330403K07Rik	RIKEN cDNA 6330403K07 gene		15.56		
43.21	70905738	Nlrp1a	NLR family, pyrin domain containing 1A	ND	ND	ND	ND
43.23	71055932	Nlrp1c	NLR family, pyrin domain containing 1C	ND	ND	ND	ND

43.62	71563422	Wscd1	WSC domain containing 1	12.54	15.54		10.70
43.89	71967431	4933427D14Rik	RIKEN cDNA 4933427D14 gene				5.38
43.95	72055496	Slc13a5	solute carrier family 13 (sodium-dependent citrate transporter), member 5				21.56
44.0	72127946	Fbxo39	F-box protein 39	85.48	57.51	27.71	55.02
44.03	72158224	Tekt1	tektin 1		11.03		
**44.15**	**72202666**	**Smtnl2**	**smoothelin-like 2**				**4.73**
**44.27**	**72249028**	**Ggt6**	**gamma-glutamyltransferase 6**				**41.01**
**44.31**	**72265140**	**Spns2**	**spinster homolog 2 (Drosophila)**			**45.78**	
**45.25**	**73080890**	**Trpv3**	**transient receptor potential cation channel, subfamily V, member 3**	**9.02**			
45.28	73118494	Aspa	aspartoacylase	7.27	5.24	39.12	25.02
**45.76**	**74487451**	**Pafah1b1**	**platelet-activating factor acetylhydrolase, isoform 1b, subunit 1**		**5.23**		
45.76	74584332	Mett10d	methyltransferase 10 domain containing				9.15
